# HIV testing, test results and factors influencing among infants born to HIV positive mothers in public hospitals of Mekelle City, North Ethiopia: a cross-sectional study

**DOI:** 10.1186/s12879-020-4790-9

**Published:** 2020-01-21

**Authors:** Hiluf Ebuy, Alemayehu Bekele, Getachew Redae

**Affiliations:** 1 Department of Midwifery, Mekelle University, College of Health Science, Ayder Comprehensive Specialized Hospital, Mekelle, Tigray Ethiopia; 2 Ethiopian Public Health Association, Addis Ababa, Ethiopia; 3 College of Health Science, School of Public Health, Mekelle University, Mekelle, Tigray Ethiopia

**Keywords:** HIV exposed infants, Timely infant testing, Associated factors, HIV positivity

## Abstract

**Background:**

Timely infant testing for HIV is critical to ensure optimal treatment outcomes among exposed infants. While world health organization recommends HIV exposed infants to be tested between 4 to 6 weeks of age, in developing countries like Ethiopia, access to timely infant testing is still very limited. The study is intended to assess timely infant testing, testing for HIV at the 18th month, test results and factors influencing HIV positivity among infants born to HIV positive mothers in public hospitals of Mekelle, Ethiopia.

**Methods:**

A cross-sectional study design was employed on 558 HIV exposed infants, using consecutive sampling technique. A checklist was used to extract 4 years (January 2014–December 2017) secondary data, collected from January–April 2018. Data were analyzed using SPSS version 20, and binary logistic regression model was used to examine the association of independent variables with the outcome variables.

**Results:**

Timely infant testing for HIV accounted for 346(62.0%). Mothers who attended antenatal care (AOR: 2.77; 95% CI: 1.17, 6.55) and who were counselled on feeding options (AOR: 2.01; 95% CI: 1.11, 3.65) were strongly associated with timely infant testing. Poor maternal adherence status was associated with infants’ HIV positivity at the 18th month of antibody test (AOR: 15.93; 95% CI: 2.21, 94.66). Being rural resident (AOR: 4.0; 95% CI: 1.23, 13.04), being low birth weight (AOR: 5.64; 95% CI: 2.00, 16.71) and not receiving ARV prophylaxis (AOR: 4.70; 95% CI: 1.15, 19.11) were positively associated with the overall HIV positivity.

**Conclusions:**

A considerable proportion of exposed infants did not undergo timely testing for HIV. Antenatal care follow-up and counselling on feeding options were associated with timely infant testing. Mother’s poor adherence status was associated with infant’s HIV positivity at the 18th month of antibody testing. Being rural resident, being low birth weight, and not receiving ARV prophylaxis were the factors that enhance the overall HIV positivity. Timely infant testing, counselling on feeding options and adherence should be intensified, and prevention of mother-to-child transmission program in rural settings need to be strengthened.

## Background

There are many ways of HIV transmission. One way of transmission is from an HIV positive woman to her child during pregnancy, labor, and breastfeeding. It is also called vertical transmission which accounts for 14% of all new HIV infections worldwide. In developing countries, despite the availability of proven interventions for the Prevention of Mother-to-Child Transmission (PMTCT), pediatric HIV is still a largely uncontrolled epidemic [1–3].

Timely infant testing for HIV infection is critical to ensure optimal treatment outcomes among exposed infants. Without testing and effective treatment, one-third of HIV-positive infants will die before the age of one year, and almost half by their second year of life. While world health organization (WHO) recommends HIV exposed infants to be tested between four to six weeks of age, in developing countries access to timely infant testing is still very limited[4, 5].

Globally, in 2015, only 43% of infants exposed to HIV during pregnancy were tested within the recommended period of time. Majority of the non-timely tested infants were from developing countries, particular Sub-Saharan Africa. In Sub-Saharan Africa, delayed infant testing is emerging as one of the challenging complex issues facing children infected with and affected by HIV. Similar to the other Sub-Saharan African coutries, studies from Ethiopia also showed that timely infant testing for HIV is very low [6–9].

The prevalence of HIV among exposed infants can reach up to 45% if left without PMTCT interventions. Nearly two-third of pregnant women living with HIV in the Middle East and North Africa passed the virus onto their infants in the year 2015 alone 10, 11]. In Ethiopia, vertical transmission, which accounted for more than 90% of pediatric HIV, is a very critical issue. Accordingly, HIV related estimates and projections showed that the national estimate of MTCT rate was 25% in 2013 and 17% in 2015. This high magnitude makes HIV/AIDS one of the top priorities of the Health Sector Transformation Plan (HSTP) of Ethiopia [12–15].

Although timely HIV testing of infants is not yet optimal, some strategies and solutions have proven successful, including community-based interventions and support and education of mothers. The Joint United Nations Program on HIV/AIDS (UNAIDS) 2016–2021 strategy set about ten targets to end the AIDS epidemic by 2030; one of the targets is to make new HIV infections among children zero and improve the health and wellbeing of mothers. The global plan to eliminate new HIV infections among children and improve the health of mothers targets to reduce the MTCT rate to less than 5% among breast feeding population and to less than 2% among non-breast feeding population [5, 16, 17].

Pertaining to the factors for HIV positivity of exposed infants, different studies reported various determinants. Accordingly, mothers with high viral load, symptomatic disease, failure to use ARV drugs during pregnancy, vaginal delivery, rupture of membrane > 4 h, low birth weight babies, premature births, and infants’ failure to use Antiretroviral (ARV) prophylaxis were found to be associated with HIV positivity among infants [18–20].

In most resource-limited countries, Deoxyribonucleic Acid Polymerase Chain Reaction (DNA/PCR) test is not available for the timely testing of exposed infants, which plays a big role in infants’ late initiation of treatment which then leads to HIV related pediatric mortality. In Ethiopia, previous studies tried to demonstrate exposed infants’ HIV testing and determinants of PMTCT. However, most of the studies were confined to a single health facility and only tried to find out factors associated with HIV positivity at 6 weeks of testing.

There is paucity of information regarding the factors affecting timely infant testing and HIV positivity at the 18th month of antibody test, until the infant completes the program. Thus, the present study is intended to assess timely infant testing, testing for HIV at the 18th month, test results and factors influencing overall HIV positivity and HIV positivity at 18 months among infants born to HIV positive mothers in public hospitals of Mekelle city, North Ethiopia.

## Methods

### Study setting

This study was conducted in Mekelle city. Mekelle is the capital city of the Tigray region and is located around 783 km north of Addis Ababa, the capital city of Ethiopia. There are three public hospitals in the city namely; Ayder Comprehensive Specialized, Quiha and Mekelle General Hospitals. The College of Health Sciences (CHS), Ayder Comprehensive Specialized Hospital (ACSH) is a public hospital with a capacity of 500 inpatient beds and has the responsibility of rendering patient care, training for medical & health science students and also carrying out research. The institution has a clinic dedicated for maternal, neonatal and child health services (MNCH) including PMTCT services.

Quiha General Hospital, which was established in 1985 by Italian cooperation, served as a health center until 2012 and then become a general hospital. Currently, the hospital has a capacity of 50 beds and the HIV care and treatment clinic is one of the core parts of the hospital’s activities. Mekelle General Hospital, which was established in 1962, is the oldest general hospital in the region. It has its own separate fully functional ART clinic and PMTCT services. Since there was no access to the DNA/PCR test in all the public hospitals, the test was done in the regional laboratory after DBS was collected in the public hospitals. On the other hand, antibody test was conducted in the study setting [21–23].

### Study design and period

A cross-sectional study design was employed in the Public Hospitals of Mekelle city from January 2014–December 2017.

### Study population, sample size and sampling procedure

The study participants consisted of all mother-infant pairs who were eligible to be enrolled to the PMTCT program in the Public Hospitals of Mekelle city during the study period. Four years data were extracted retrospectively, from January 2014–December 2017. The reason why we started extracting data from 2014 was due to the fact that in Ethiopia option B plus strategy for PMTCT was adopted in 2013 [24]. However, in the study area consistent and full documentation of option B plus services were available starting 2014. In the four years period, a total of 558 mother-infant pairs who fulfilled the inclusion criteria were enrolled to the study, using consecutive sampling technique. We used consecutive sampling technique because it was a secondary data, 30% of the participants were excluded from the study due to either incomplete information, loss to follow up or transfer out.

All mother-infant pairs who sought and completed the PMTCT program were included in the study. We excluded 57 participants who had an incomplete medical records, 148 lost to follow up subjects and 34 participants who were transferred into other health facilities. Loss to follow up, which makes identifying and managing HIV-infected children very difficult, could be a reflection of failure in PMTCT.

### Data collection procedure

After reviewing different literatures [8, 9, 25, 26], a checklist developed by the authors was used to collect data regarding socio-demographic and health-related factors. Data were extracted from secondary data sources retrospectively by reviewing patient charts (both paper and electronic medical recording systems), PMTCT, antenatal, delivery, and postnatal care registration books and partographs from January–April, 2018.

### Operational definitions

#### Timely infant testing

There is a special HIV detection test done for HIV exposed infants after Dried Blood Spot (DBS) is collected called DNA/PCR test. This HIV testing is recommended to be done within 4–6 weeks of age and is referred to as timely infant testing.

#### Overall rate of MTCT

This is the aggregate of MTCT of HIV at the 6th week of DNA/PCR test and the 18th month of antibody test, until weaning.

#### Good adherence

Ratios of ART medication adherence equal to or greater than 95% were defined as good dosage adherence. Adherence status of the participants was measured from a self-report.

### Data quality control

The checklist was prepared in English and reviewed by senior researchers and feedback was incorporated accordingly. The secondary data were extracted by three midwives, one for each hospital and training was given concerning the checklist, data collection technique, purpose of the study, and keeping confidentiality. The research team had day to day on-site supervision to make sure data extraction from secondary sources was going smoothly. The research team discussed how to take corrective measures like checking and discarding data if an error, such as illegibly documented checklists, occurred during the data collection. The collected data were checked for completeness, consistency, and clarity.

### Data management and analysis

The collected data were coded and entered into an excel spreadsheet; it was then exported to Statistical Package for Social Sciences (SPSS) version 20 statistical software for analysis. Tables and figures were used for descriptive statistics. Frequency and percentage were used for categorical variables. Odds ratio (OR) with 95% confidence interval was used to measure strength of the association. Binary logistic regression model, with bivariate and multivariable analysis, was used to examine the association of independent variables with the outcome variables and calculate their crude as well as adjusted odds ratios.

Those variables which happened to have a *p*-value < 0.25 with crude analysis of logistic regression model were fitted into the final multivariable logistic regression model and their adjusted odds ratios were calculated. Statistical significance was declared using an odds ratio and 95% confidence interval. Absence of multicollinearity was checked and found to be satisfied with the maximum variance inflation factor (VIF) value of 1.8. Goodness of fit was checked by Hosmer-Lemeshow test which yielded a chi-square value of 1.4 with 8 df and *p*-value of 0.99 for the final model of overall HIV positivity.

### Ethical considerations

Ethical clearance was obtained from the institutional review board of the Ethiopian Public Health Association (EPHA). The collected data was kept anonymous; mothers and infants’ names were not recorded but instead were written down in code and additional measures like keeping the completed checklist in a safe place were taken. This study did not impose any harm to the community or to the research team, and it was conducted in line with national and international ethical guidelines. A letter of collaboration was also obtained from the institutional review board of the College of Health Sciences, Ayder Comprehensive Specialized, Quiha and Mekelle General Hospitals.

## Results

### Socio-demographic characteristics of the study participants

Among the total 797 mother-infant pairs who were enrolled in the PMTCT cohort over the past four years, 558(70.0%) of them fulfilled the inclusion criteria and were included in the final analysis. Two hundred thirty-nine (30.0%) participants were excluded from the study, of which 57(23.85%) had an incomplete medical record, 148(61.95%) were lost to follow up and the remaining 34(14.2%) were transferred into other health facilities. The mothers’ ages ranges from 15 to 49 years; with a mean age of 28.89 years and a standard deviation of 4.65 years. Regarding residence, the majority of mothers, 459(82.3%), were urban dwellers. There were 291(52.2%) female HIV exposed infants (Table 1)

**Table 1:** Socio-demographic characteristics of the study participants in Public Hospitals of Mekelle city, Tigray, Ethiopia, 2014–2017 (*N* = 558)

Variable	Category	Frequency	Percent
Health institution where PMTCT services provided	ACSH	98	17.6
	Quiha Hospital	142	25.4
	Mekelle General Hospital	318	57.0
Age of mothers (years)	15–24	99	17.7
	25–34	360	64.6
	35–49	99	17.7
Residence	Urban	459	82.3
	Rural	99	17.7
Mother had a partner	Yes	499	89.4
	No	59	10.6
Sex of infants	Female	291	52.2
	Male	267	47.8

ACSH Ayder Comprehensive Specialized Hospital

### PMTCT service and clinical characteristics among mothers

The majority (95.0%) of the mothers had attended ANC and 529(94.8%) mothers were on ART on entry to the PMTCT cohort. Of the 513(91.4%) mothers whose adherence status was known, 312(60.8%) had good adherence status. WHO clinical staging was documented in the majority (95.7%) of mothers and 505(94.6%) of them were in clinical stage I or II (Table 2).

**Table 2:** PMTCT service and clinical characteristics among mothers in Public Hospitals of Mekelle city, Tigray, Ethiopia (*N*= 558)

Variable	Category	Frequency	Percent	95% CI
Had ANC follow up	Yes	530	95.0	95.5, 98.4
	No	28	5.0	3.2, 7.0
Mother on ART on entry to PMTCT cohort	Yes	529	94.8	93.0, 96.8
	No	29	5.2	3.4, 7.2
Mothers current ART drug regimen	TDF-3TC-EFV	386	69.2	65.0, 73.1
	AZT-3TC-EFV	38	6.8	4.8, 9.3
	AZT-3TC-NVP	107	9.2	15.8, 22.6
	TDF-3TC-NVP	27	4.8	3.2, 6.8
Mothers CD4 count done	Yes	531	95.2	92.9, 96.8
	No	27	4.8	3.2, 7.1
Current CD4 count of mothers	< 500 mm^3^	204	38.4	33.9, 42.4
	≥500 mm^3^	327	61.6	57.6, 66.1
Mothers Adherence status known	Yes	513	91.4	87.6, 93.1
	No	45	8.6	6.9, 12.4
Mothers adherence status	Good	312	60.8	56.3, 65.1
	Fair	183	35.7	31.6, 40.2
	Poor	18	3.5	2.1, 5.3
Mothers WHO clinical staging done	Yes	534	95.7	93.1, 97.8
	No	24	4.3	2.2, 6.9
Mothers WHO clinical stage	Stage I / II	505	94.6	92.7, 96.4
	Stage III / IV	29	5.4	3.6, 7.3
Mothers counselled on feeding options	Yes	493	88.4	85.7, 91.0
	No	65	11.6	9.0, 14.3
Provision of postpartum family planning	Yes	301	53.9	41.4, 50.4
	No	257	46.1	49.6, 58.6

### PMTCT interventions for infants and other clinical characteristics

The mean weight of the infants was 3.1 kg, with a standard deviation of 0.36 kg. Eighty-four (15.1%) infants’ were low-birth-weights. The majority (94.3%) of the infants were given ARV prophylaxis. Regarding the feeding of exposed infants, no mixed feeding was practiced. Timely infant testing for HIV accounted for 346(62.0%) (Table 3).

**Table 3:** PMTCT interventions by infants and other clinical characteristics in Public Hospitals of Mekelle city, Tigray, Ethiopia (*N* = 558)

Variable	Category	Frequency	Percent	95% CI
Infant ARV prophylaxis (NVP syrup) given	Yes	526	94.3%	92.3, 96.1
	No	32	5.7%	3.9, 7.7
Infants birth weight	Normal birth-weight	474	84.9	81.7, 88.0
	Low birth-weight	84	15.1	12.0, 18.3
Infants feeding practice	EBF	432	87.6	84.8, 90.7
	EFF	61	12.4	9.3, 15.2
Timely infant testing	Yes	346	62.0	58.4, 66.3
	No	212	38.0	33.7, 41.6
Partner test result	Reactive	246	49.3	44.7, 53.3
	Non-reactive	53	10.6	7.8, 13.2
	Not determined	200	40.1	36.1, 44.5
Mode of delivery	SVD	432	77.4	74.0, 80.5
	C/section	126	22.6	19.5, 26.0
Place of delivery	Health institution	548	98.2	97.1, 99.1
	Home	10	1.8	0.9, 2.9
Rupture of membrane for more than 4 h	Yes	119	21.3	18.1, 24.4
	No	439	78.7	75.6, 81.9

### Mother-to-child transmission rate of HIV

Overall 558 study participants were tested for DNA/PCR. Of these, 13(2.3%) (95% CI: 1.3, 3.8%) tested positive for HIV. Among the 545 HIV exposed infants for whom the rapid antibody test was done, 7(1.3%) (95% CI: 0.4, 2.4%) tested positive for HIV. Therefore, the overall MTCT rate was 3.6% (95% CI: 2.2, 5.4%).

### Factors associated with timely infant testing of HIV

In the multivariable logistic regression analysis, factors found to be significantly associated with timely infant testing were ANC follow up and feeding option counselling. The odds of practicing timely infant testing of mothers who attended ANC follow up were 2.77 times higher than those who didn’t have ANC follow up (AOR: 2.77; 95% CI: 1.17, 6.55). The odds of practicing timely infant testing of infants whose mothers were counselled on feeding options were 2-fold higher than their counterparts (AOR: 2.01; 95% CI: 1.11, 3.65)(Table 4).

**Table 4:** Factors associated with timely infant testing of HIV in Public Hospitals of Mekelle city, Tigray, Ethiopia (*N* = 558)

Variable	Category	COR (95% CI)	AOR (95% CI)
Place of residence	Rural	1	1
	Urban	1.32(0.85, 2.05)	1.15(0.68, 1.95)
Had ANC follow up	No	1	1
	Yes	2.65(1.22, 5.78)*	2.77(1.17, 6.55)*
Mothers WHO clinical stage	Stage III / IV	1	1
	Stage I / II	1.56(0.74, 3.30)	1.49(0.66, 3.35)
Current CD4 count of mothers	< 500 mm^3^	1	1
	≥500 mm^3^	1.14(0.79, 1.63)	1.36(0.91, 2.04)
Mothers adherence status	Poor	1	1
	Fair	1.91(0.72, 5.04)	1.15(0.38, 3.46)
	Good	1.52(0.57, 3.93)	0.94(0.32, 2.81)
Provision of postpartum family planning	No	1	1
	Yes	0.98(0.67, 1.38)	1.09(0.74, 1.61)
Infants birth weight	Low birth-weight	1	1
	Normal birth-weight	1.27(0.79, 2.03)	0.78(0.44, 1.39)
Mothers counselled on feeding options	No	1	1
	Yes	1.81(1.08, 3.04)*	2.01(1.11, 3.65)*
Infants ARV prophylaxis (NVP syrup) given	No	1	1
	Yes	1.12(1.54, 2.33)*	2.08(0.89, 4.88)

*COR* Crude odds ratio, *AOR* Adjusted odds ratio *Significant association *CI* Confidence interval

### Factors associated with HIV positivity at the 18th month

At the 18th month of antibody test the mother’s adherence status was the only variable that had a significant association with HIV positivity. The odds of HIV positivity of infants whose mother’s adherence status was poor were 15.93 times higher than those with good adherence status (AOR: 15.93; 95% CI: 2.21, 94.66) (Table 5).

**Table 5:** Factors associated with HIV positivity at 18th month among exposed infants in Public Hospitals of Mekelle city, Tigray, Ethiopia (*N* = 558)

Variable	Category	COR (95% CI)	AOR (95% CI)
Place of residence	Urban	1	1
	Rural	3.68(0.81, 16.74)	3.45(0.63, 18.78)
Mother on ART on entry to PMTCT cohort	Yes	1	1
	No	3.15(0.46, 27.16)	1.47(0.12, 17.41)
Mothers WHO clinical stage	Stage I / II	1	1
	Stage III / IV	3.13(0.36, 26.95)	1.05(0.06, 19.37)
Mothers adherence status	Good	1	1
	Fair	0.59(0.06, 5.69)	0.60(0.06, 6.12)
	Poor	20.0(3.78, 99.33) *	15.93(2.21, 94.66) *
Provision of postpartum family planning	Yes	1	1
	No	1.52(0.39, 6.88)	1.18(0.19, 7.39)
Infants birth weight	Normal birth-weight	1	1
	Low birth-weight	4.70(01.03, 21.45) *	4.40(0.82,23.66)
Infants feeding practice	EFF	1	1
	EBF	0.84(0.11, 7.09)	0.66(0.06, 7.17)

COR Crude odds ratio, *AOR* Adjusted odds ratio *Significant association *CI* Confidence interval

### Factors associated with overall HIV positivity

In the multivariable logistic regression, the variables; place of residence, infants’ birth weight, and infants’ ARV prophylaxis had a significant association with overall HIV positivity among exposed infants. Accordingly, the likelihood of HIV positivity among rural residents was 4 times higher than urban dwellers (AOR: 4.00; 95% CI: 1.23, 13.04). The odds of HIV positivity of low birth-weight infants were 5.64 times higher than normal birth-weight infants (AOR: 5.64; 95% CI: 2.00, 16.71). The likelihood of HIV positivity among infants who did not receive ARV prophylaxis was 4.7-fold higher than their counterparts (AOR: 4.70; 95% CI: 1.15, 19.11) (Table 6).

**Table 6:** Factors associated with overall HIV positivity among exposed infants in Public Hospitals of Mekelle city, Tigray, Ethiopia (*N* = 558)

Variable	Category	COR (95% CI)	AOR (95% CI)
Place of residence	Urban	1	1
	Rural	3.27(1.30, 8.24) *	4.00(1.23, 13.04) *
Had ANC follow up	Yes	1	1
	No	3.62(1.00, 13.17) *	2.06(0.35, 12.11)
Mother on ART on entry to PMTCT cohort	Yes	1	1
	No	2.10(0.46, 9.53)	2.56(0.36, 17.98)
Mothers WHO clinical stage	Stage I / II	1	1
	Stage III / IV	3.31(0.91, 12.02)	2.14(0.47, 9.87)
Current CD4 count of mothers	≥500 mm^3^	1	1
	< 500 mm^3^	1.63(0.64, 4.18)	1.83(0.61, 5.50)
Mothers adherence status	Good	1	1
	Fair	2.52(0.94, 6.75)	1.76(0.57, 5.47)
	Poor	8.71(2.05, 37.10) *	1.04(0.07, 16.06)
Provision of postpartum family planning	Yes	1	1
	No	0.49(0.19, 1.29)	0.45(0.13, 1.53)
Mode of delivery	C/Section	1	1
	SVD	1.68(0.48, 5.83)	2.10(0.49, 9.01)
Infants birth weight	Normal birth-weight	1	1
	Low birth-weight	6.27(2.52, 15.58) *	5.64(2.00,16.71) *
Infants feeding practice	EFF	1	1
	EBF	0.79(0.25, 2.79)	1.17(0.24, 5.76)
Infants ARV prophylaxis (NVP syrup) given	Yes	1	1
	No	4.55(1.47, 14.52) *	4.70(1.15, 19.11) *

COR Crude odds ratio, AOR Adjusted odds ratio *Significant association *CI* Confidence interval

## Discussion

The present study was intended to assess timely infant testing, testing for HIV at the 18th month, test results and factors influencing overall HIV positivity and HIV positivity at 18 months among infants born to HIV positive mothers in public hospitals of Mekelle, Tigray, Ethiopia.

The percentage of HIV exposed infants tested for HIV timely (62.0%) in our study were comparatively higher than studies conducted in South Gondar, Ethiopia in 2014(25.3%) and Asella Teaching and Referral Hospital, Ethiopia from 2012 to 2015(19.2%) [8, 9]. This high percentage of timely infant testing in our study could be due to the fact that the regional laboratory, where the DNA/PCR test is done, is located in the same city where the study is conducted, which makes it easier for the DNA/PCR test to be done in a timely manner.

In the first six weeks the MTCT rate of HIV, as determined by a DNA/PCR test, was 2.3%(95% CI: 1.3, 3.8%). This was one of the lowest MTCT rates of reports compared to a cross-sectional household survey conducted in 26 communities across Zambia, South Africa, Cote d’Ivoire, and Cameroon in 2015(11.0%). This can be explained by the fact that our study was an institutional-based study while the household survey was a community-based study. This study’s MTCT rate of DNA/PCR test result was also less than studies conducted in Addis Ababa, Ethiopia in 2014(6.0%), Southwest, Ethiopia in 2014(9.6%), South Gondar, Ethiopia in 2014(10.1%) and Arsi Ethiopia between 2012 & 2015 (3.1%) [8, 9, 26–28]. This could be attributed to the reason that almost all of our study participants gave birth in a health institution, which allowed them good access to the first six weeks of a PMTCT program.

In our study, the overall MTCT rate of HIV infection among exposed infants was 3.6% (95% CI: 2.2, 5.4%), which was much less than a study conducted in Brazil. A possible explanation for this might be that the study conducted in Brazil was a 21 years study, which started when PMTCT program wasn’t as efficient and modernized as recently. Our finding was also less than Ethiopia’s national estimate of MTCT in 2013 and 2015 and from studies done in Arsi add Dire Dawa, Ethiopia [9, 12, 13, 20, 25]. This may be explained by the reason that no mixed feeding was practiced in our study, which enhances the odds of HIV positivity. In addition, the majority of our participants were from urban areas where they can easily access PMTCT services, while the national estimates account for the overall MTCT of the country, including rural residents and home deliveries.

Mothers who attended ANC follow up through the course of the pregnancy were 2.77 times more likely to practice timely infant testing of HIV compared to the mothers who didn’t have ANC follow up. The conceivable reason could be that one of the components of PMTCT services during ANC is health education and awareness creation about the advantages of timely infant testing. This was given to the mothers who had ANC follow up when they visited health institutions.

Counselling on feeding options was another factor associated with timely infant testing. The odds of practicing timely infant testing of infants whose mothers were counselled on feeding options were 2-fold higher than mothers of infants who weren’t counselled on feeding options. This could be attributed to the reason that when mothers were counselled on feeding options, at the same time, they were also being counselled on other PMTCT cascades, such as advantages of timely infant testing.

Mother’s adherence status had a significant association with HIV positivity among exposed infants at the 18th month rapid antibody test. Infants born to HIV positive mothers whose adherence status was poor were 15.93 times at higher risk of acquiring HIV infection than infants born to HIV positive mothers whose adherence status was good. The possible risk difference could be due to the fact that poor maternal adherence status causes drug resistance, which then leads to the elevation of maternal viral load, putting exposed infants at high risk of HIV positivity.

The odds of HIV positivity of infants born to HIV positive mothers from rural residences were 4 times higher than infants born to mothers of urban residents. This is consistent with the finding of studies conducted in Dil Chora Hospital in Dire Dawa, Ethiopia and Gondar University Hospital in northwest Ethiopia [25, 29]. A likely explanation is that mothers from rural settings might have limited access to ANC clinics and therefore poor access to PMTCT services compared to mothers who are urban dwellers.

The odds of overall HIV positivity of low birth-weight infants were 5.64 times higher than the normal birth-weight infants. This is consistent with researches done in Arsi and Addis Ababa, Ethiopia[9, 26]. The attributed risk difference could be due to the reason that the immature gastrointestinal tract in low birth weight infants might facilitate MTCT of HIV through mother’s breast milk.

ARV prophylaxis for infants was another factor associated with exposed infants’ overall HIV positivity. The odds of acquiring HIV among infants who did not receive ARV prophylaxis was 4.7-fold higher than those infants who received ARV prophylaxis. The observed risk difference could be due to the reason that ARV prophylaxis for exposed infants decreases vertical transmission of the virus from HIV positive mothers. This finding is in tune with studies conducted in Brazil and Ethiopia[20, 25, 26, 30].

### Strength of the study

This study gives a clue about the factors associated with timely infant testing and potential factors associated with HIV positivity among exposed infants. Furthermore, the findings of the study are meant to strengthen and enhance the implementation of the PMTCT program at the hospital level in the public health system.

### Limitations of the study

This study has some limitations. The fact that it is a cross-sectional study design is one of the study’s weakness. Since the study used secondary data, the information gathered was incomplete. As a result, some sociodemographic variables such as household income and educational status were not documented. Additionally, this study didn’t specify the time when infants’ ARV prophylaxis was initiated.

## Conclusions

In conclusion, considerable proportion of exposed infants did not undergo timely infant testing for HIV. There was relatively low MTCT rate of HIV at six weeks, eighteen months and for overall HIV testing. Antenatal care follow-up and counselling on feeding options were associated with timely infant testing. Mother’s poor adherence status was associated with infant’s HIV positivity at the 18th month of antibody test. Infants born to HIV positive mothers from rural residences, low birth-weights and who didn’t receive ARV prophylaxis after birth were the factors that enhance the risk of overall HIV positivity.

### Recommendations

Health care providers should work on strengthening timely testing of HIV among exposed infants. Counseling on feeding options, ANC and adherence needs to be provided in a sustainable way. Continuous follow up of exposed infants has to be intensified. In addition, policymakers and program managers should focus on scaling up PMTCT program in rural settings. A prospective study need to be conducted to assess the effectiveness of PMTCT programs and to address additional factors associated with HIV positivity among exposed infants.

## Data Availability

The datasets generated and/or analyzed during the current study are not publicly available due to ethical and confidentiality reasons but are available from the corresponding author on reasonable request under the Ethics Committee’s approval.

## Abbreviations

ACSH: Ayder Comprehensive Specialized Hospital
AIDS: Acquired Immunodeficiency Syndrome
ANC: Antenatal Care
ART: Antiretroviral Therapy
ARV: Antiretroviral
CD4: Cluster of Differentiation 4
CS: Caesarean Section
DBS: Dried Blood Spot
DNA/PCR: Deoxyribonucleic Acid Polymerase Chain Reaction
EBF: Exclusive Breast Feeding
EFF: Exclusive Formula Feeding
EPHA: Ethiopian Public Health Association
HIV: Human Immunodeficiency Virus
HSTP: Health Sector Transformation Plan
LSI: Leadership in Strategic Information
MNCH: Maternal, Neonatal and Child Health
MTCT: Mother -To-Child Transmission
OR: Odds ratio
PMTCT: Prevention of Mother-to-Child Transmission
SPSS: Statistical Package for Social Science
UNAIDS: Joint United Nations Programme on HIV/ AIDS
WHO: World Health Organization
